# Chlorogenic acid attenuates liver apoptosis and inflammation in endoplasmic reticulum stress-induced mice

**DOI:** 10.22038/IJBMS.2023.66827.14659

**Published:** 2023-04

**Authors:** Azam Moslehi, Tahereh Komeili-Movahhed, Mostafa Ahmadian, Mahdieh Ghoddoosi, Fatemeh Heidari

**Affiliations:** 1 Cellular and Molecular Research Center, Qom University of Medical Sciences, Qom, Iran

**Keywords:** Apoptosis, Chlorogenic acid, Endoplasmic reticulum – stress, Inflammation, Steatosis

## Abstract

**Objective(s):**

The accumulation of unfolded or misfolded proteins in the endoplasmic reticulum (ER)results in a state known as “ER stress”. It can affect the fate of proteins and play a crucial role in the pathogenesis of several diseases. In this study, we investigated the protective effect of chlorogenic acid (CA) on the inflammation and apoptosis of tunicamycin-induced ER stress in mice.

**Materials and Methods:**

We categorized mice into six groups: Saline, Vehicle, CA, TM, CA 20-TM, and CA 50-TM. The mice received CA (20 or 50 mg/kg) before intraperitoneal tunicamycin injection. After 72 hr of treatment, serum biochemical analysis, histopathological alterations, protein and/or mRNA levels of steatosis, and inflammatory and apoptotic markers were investigated by ELISA and/or RT-PCR.

**Results:**

We found that 20 mg/kg CA decreased mRNA levels of *Grp78, Ire-1*, and *Perk*. Moreover, CA supplementation prevented TM-induced liver injury through changes in lipid accumulation and lipogenesis markers of steatosis (*Srebp-1c, Ppar-α, and Fas*), and exerted an inhibitory effect on inflammatory (*NF-κB, Tnf-α, *and* Il-6*) and apoptotic markers (caspase 3, *p53*, *Bax*, and *Bcl2*), of liver tissue in ER stress mice.

**Conclusion:**

These data suggest that CA ameliorates hepatic apoptosis and inflammation by reducing NF-κB and Caspase 3 as related key factors between inflammation and apoptosis.

## Introduction

The endoplasmic reticulum (ER) is a multifunctional organelle in which the folding of newly synthesized secretory and membrane proteins, lipid biosynthesis, and calcium storage occur (1). 

Misfolding of some proteins occurs during biosynthesis in the ER lumen. The accumulation of unfolded or misfolded proteins in the ER results in a state known as “ER stress”. It can affect the fate of proteins, lipids, and carbohydrates, lead to inflammatory signaling in the stressed cells, cause cellular apoptosis, and promote disease processes (2). Cross-talk between ER stress and inflammation has been demonstrated in many pathological conditions. Increasing evidence has demonstrated that ER stress is associated with chronic diseases, including diabetes and obesity, multiple forms of respiratory inflammation, neuromuscular and neurodegenerative inflammatory diseases, arthritis, autoimmune disease, inflammatory bowel diseases, cancer, ischemia, and liver fat accumulation disease (hepatic steatosis) (3-6).

Some prevalent factors such as oxidative stress, viral infections, dietary demands, and pharmacologic stimuli (7) induce ER stress by altering the redox state, calcium levels, or failure to modify secretory proteins post-translationally. Pharmacologically, toxins such as tunicamycin (TM) and thapsigargin inhibit protein glycosylation and disrupt ER Ca^2+^ levels leading to ER stress (8). Since sustained or massive ER stress leads to apoptosis, if the stress cannot be resolved, it will be lethal to cells, and signaling switches to a pro-apoptotic response by influencing unfolded protein response mediated signals (9). 

As mentioned above, inflammation plays an important role in creating ER stress. On the other hand, the evidence from available literature shows that a variety of plants and their derived bioactive combinations have distinctive properties that permit them to act as potent anti-inflammatory compounds (10), such as chlorogenic acid (CA) (11). 

CA (5-O-caffeoylquinic acid), a polyphenolic compound widely distributed in foods and herbs, is one of the main polyphenols in the human diet, and it takes many health-promoting properties (12). Foods and herbs such as tomatoes (13), potatoes, pears, tobacco leaves, apples, eggplants, coffee beans, honeysuckle (13, 14), artichoke (15), grapes (16), plums (13, 17), kiwi fruit (18), and tea (16, 19) contain a significant amount of CA. Recent studies demonstrated that CA has anti-inflammatory (11), anti-oxidant (11), anti-diabetic (20), anti-cancer (21), anti-neurodegenerative (22), anti-lipidemic (20) and anti-hypertensive activities (23). 

According to the beneficial effects mentioned above, we evaluated the effects of CA administration on liver steatosis, inflammation, and apoptosis in TM-induced ER stress. 

## Materials and Methods

***Chemicals and reagents***

CA (C3878, purity ≥ 95%) was purchased from Sigma-Aldrich (St. Louis, MO, USA). TM was purchased from Cayman Chemical (Ann Arbor, MI, USA). TM was prepared in dimethyl sulfoxide (DMSO; Calbiochem, EMD Bioscience Inc. La Jolla, CA, USA).

***Animals***

In the present study, 36 male C57/BL6 mice weighing 22-25 g were purchased from the Pasteur Institute (Tehran, Iran). The study commenced after obtaining the approval of the experimental animal ethics committee (Ethics code: IR.MUQ.REC.1400.056). Mice were provided with fresh drinking water daily and were kept at 21 ˚C with 12 hr light (08:00–20:00 hr) and 12 hr darkness (24) with free access to standard laboratory chow (Pars animal feed Co, Tehran, Iran).

***Experimental design***

Before each analysis, animals were randomly divided into 6 equal groups (n= 6), including Saline (0.2 mL sterile normal saline injection), Vehicle ( 0.2 mL DMSO injection), CA (50 mg/Kg CA injection) (25, 26), TM (a single dose of 2 µg/g TM injection to induce ER stress) (27), CA 20-TM (a single dose of 20 mg/kg CA 60 minutes before TM administration) (28), CA 50-TM (a single dose of 50 mg/kg CA 60 minutes before TM administration). Intraperitoneal administration was used for all groups.

Thirty hours post-TM injection (29), the animals were anesthetized with ketamine (100 mg/kg) and xylazine (10 mg/kg). The blood samples were taken from the heart for liver biochemical assays. Then, the abdomen was excised via a midline incision, and the liver was removed. Apart from the median lobe, the liver was dissected and half of it was fixed in 10% formalin for histopathology assessment and the other half was stored in a freezer (-80˚^C^) for molecular assessment. 

***Evaluation of total cholesterol (TC), triglyceride (TG), alanine aminotransferase (ALT), aspartate aminotransferase (AST), and alkaline phosphatase (ALP)***

Blood was directly collected from the heart by a 2 mL syringe insertion. Serum was obtained from the centrifuged blood samples (3500 rpm for 20 min). Then, TC, TG, ALT, and AST levels were determined according to the protocol provided by the colorimetric Kit (Pars Azmun, Iran) using a spectrophotometer (UNICO Instruments C., Model 1200, USA) (24). ALP level was determined in liver tissue samples according to the ALP colorimetric activity assay Kit (Cayman chemical, No. 701710, USA). 

***Tissue preparation and histopathology*** ***examination***

For the histological examination, the liver tissues were fixed in 10% formalin, dehydrated in ethanol series, cleaned in xylene series, embedded in paraffin wax, cut into 5 µm sections, mounted on glass slides, and stained with hematoxylin and eosin dye (H&E stain) according to Bancroft and Layton (30). Photomicrographs and histology examinations were taken using a light microscope (Leica DM750, Leica Microsystems, India). In all samples, the histological findings were scored based on the Kleiner *et al.* scoring system as follows: steatosis (0–3), lobular inflammation (0–3), and hepatocellular ballooning (0–2)(31). Then the histological findings as well as serum biomarker levels were interpreted by an expert pathologist blind to the experiment. The inflammatory cell infiltration, hepatocyte ballooning, and steatosis were evaluated using image analysis software (Image J, National Institute of Health, Bethesda, MD).

***Real-time reverse transcription polymerase chain reaction (Real-time RT-PCR)***

The total RNA of frozen tissue samples was isolated using the Trizole (Yekta Tajhiz, Iran) according to the manufacturer’s instructions. The quantity and purity of the RNA samples were measured by a Nanodrop spectrophotometer. Complementary DNAs (cDNA) were prepared from mRNA templates for RT-PCR using the RocketScript™ RT PreMix (BioNeer, Alameda, CA, USA). Real-time PCR analysis was performed with AccuPower® 2X GreenStar™ qPCR Master Mix (Biofact, Korea) using glyceraldehyde-3-phosphate dehydrogenase (*Gapdh*) as an internal control (3). The quantitation of data was performed using the comparative CT (∆∆CT) method (Table 1). 

***Enzyme-linked immunosorbent assay (ELISA) of inflammatory cytokines in the liver tissue***

The presence of Caspase 3 and NF-κB in the tissue supernatant was assayed with a mouse standard ELISA kit. Briefly, 100 mg of the liver tissue was weighed, homogenized, and added to 1 ml phosphate buffer. It was then centrifuged (3000-4000 rpm for 20 min), and the supernatant was collected, aliquoted, and kept at -80^ o^C. For NF-κB, 40 µl sample, 10 µl NF-κB- Ab, 50 µl of each standard, and 50 µl streptavidin-HRP were added and incubated at 37 ^o^C for 60 min. For Caspase 3, 40 µl samples, 10 µl caspase 3-Ab, 50 µl standards and 50 µl streptavidin-HRP were added and incubated at 37 ^o^C for 60 min. The following steps were similar for both assays. The plates were then washed five times with 300 µl wash buffer, then 100 µl chromogen was added and incubated for 10-20 min. Afterward, the stop solution was added and read at 450 nm within 10 min. The results were calculated based on the absorbance levels of complex cytokine-antibodies, and the units of cytokines were described as pg/ml (32).

***Statistics analysis***

All data are presented as mean ± SEM. Statistical analysis was performed using one-way analysis of variance (ANOVA) and Tukey’s *post hoc* test for multiple comparisons using the statistical software package SPSS Version 18.0 for Windows. In all analyses, the significance level was accepted as 0.05. 

## Results

***CA decreased TM-induced ER stress in mice***

**Figure 1:**
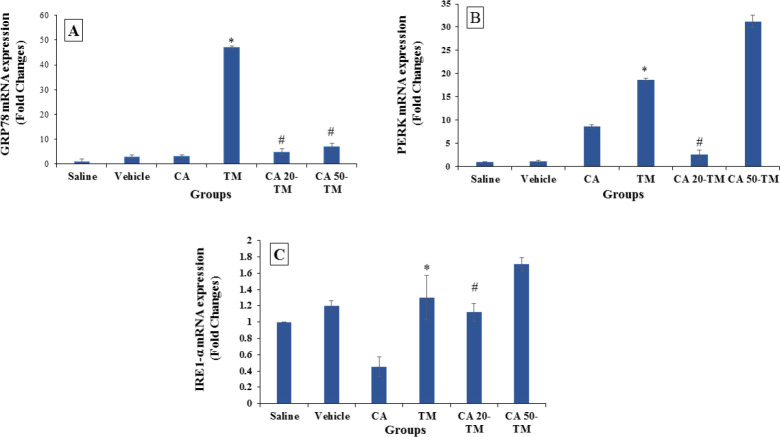
mRNA expression of Grp78, Ire1, and Perk in different experimental groups. Mean ± SEM, N = 6, *P<0.05 compared to the Saline group. #*P*<0.05 compared to the TM group (one-way ANOVA followed by Tukey’s *post hoc* test)

In this study, a single dose of TM resulted in ER stress and increased *Grp78* gene expression in the TM group compared to the saline group; but *Grp78* gene expression significantly decreased in the CA 20- TM and CA 50- TM groups compared to the TM group (*P*<0.05) (Figure 1A). 

Our finding showed that the TM injection increased the expression of *Ire-1* and *Perk* in the TM group compared to the saline group (*P*<0.05) (Figure 1B, 1C). The pretreatment with 20 mg/kg CA significantly decreased gene expression of *Ire-1* and *Perk*, while receive of 50 mg/kg CA increased gene expression compared to the TM group (*P*<0.05) (Figures 1B and 1C).

***CA ameliorated TC, TG, ALT, AST, and ALP levels on TM-induced ER stress in mice***

Blood sample analysis revealed that serum TC and TG levels in the TM group were significantly decreased compared to the saline group. Treatment with CA (both 20 and 50 mg/kg) did not increase serum TC and TG levels (*P*<0.05) (Table 2). The ALT and AST levels in serum and ALP levels in the liver tissue were measured to explore damage to the liver following TM administration. As depicted in Table 2, compared to the saline group, the TM group showed a clear increase in the levels of ALT, AST, and ALP (*P*<0.05). Mice treated with CA (20 mg/kg) experienced a significant decrease in the level of AST compared to the TM group (*P* < 0.05), but there were no significant differences in the ALT and ALP levels with the saline group (*P*>0.05). The ALT and ALP levels decreased significantly in the high concentrations of CA (50 mg/kg); however, the AST level increased significantly compared to the saline group (*P*<0.05). Interestingly both groups treated with different concentrations of CA showed lowered ALT, AST, and ALP levels compared to the TM group (*P*<0.05). 

***CA reduces steatosis and fat accumulation in the liver tissue***

**Figure 2:**
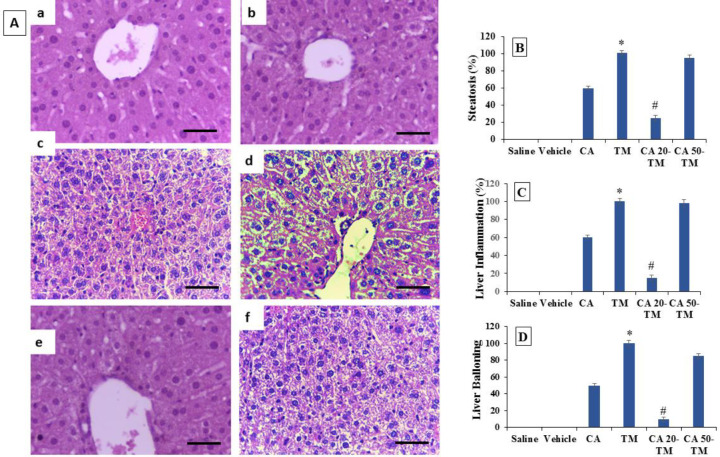
(A) Histological findings of liver tissues after H&E staining (magnification ×200) in different experimental groups. a; Saline: normal liver histology, b; Vehicle: normal liver architecture, c; CA (Chlorogenic acid): showing steatosis and ballooning degradation. d; TM: (Tunicamycin) showing steatosis and ballooning degradation (Empty spaces in the cell indicate fat accumulation and enlargement of cells), e; CA-20 TM: showing lower steatosis, inflammation, and ballooning (lower empty spaces). f; CA-50 TM: showing steatosis and ballooning. Figures (B) represent Steatosis, (C) Inflammation, and (D) Ballooning (%) (Mean ± SEM, n = 6), * compared to the Saline group, #compared to the TM group (one-way ANOVA followed by Tukey’s *post hoc *test)

In this experiment, H&E staining was performed to evaluate histopathological alterations in the liver tissue. As noted in Figure 2, no sign of steatosis or inflammation was seen in the Saline group; however, severe steatosis (excessive lipid droplet accumulation with empty spaces), severe cellular ballooning, and lobular inflammation were observed in the TM group compared to the Saline group. Nevertheless, administering 50 mg/kg CA did not decrease steatosis compared to the Saline group (Figure 2). However, in the CA 20-TM group, a clear decrease was seen in steatosis, inflammation, and cellular ballooning compared to the TM group (*P*<0.05) (Figure 2). In fact, the administration of CA (20 mg/kg) improved liver steatosis and inflammation in the ER-stress-induced mice.

***CA pretreatment alleviates steatosis in liver tissue***

**Figure 3:**
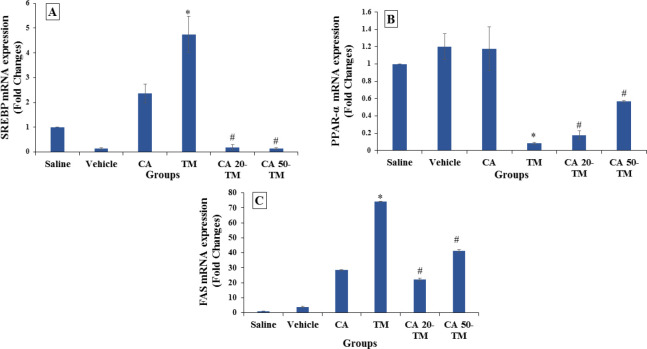
mRNA expression of *Srebp-1c*, *Ppar-α*, and *fas* in different experimental groups

The expression of the *Srebp-1c* gene in the TM group increased compared to the Saline group (*P*<0.05) (Figure 3A). This suggests that the administration of the TM caused ER stress conditions in mice. Additionally, our results showed that the level of the *Srebp-1c* transcription factor gene in groups treated with CA (20 or 50 mg/kg) was significantly downregulated compared to the TM group (*P* < 0.05). 

The level of *Ppar-α* gene expression in the TM group decreased significantly compared to the saline group (*P*<0.05). While the administration of CA (20 or 50 mg/kg) increased the level of the *Ppar-α* gene compared to the TM group (*P<*0.05) (Figure 3B).

Mice treated with the TM experienced a significant increase in the levels of *Fas* compared to the saline group, but it significantly decreased in CA (20 and 50 mg/kg) treated mice compared to the TM group (*P*<0.05) (Figure 3C). 

***CA pretreatment changes pro-inflammatory cytokines***

**Figure 4:**
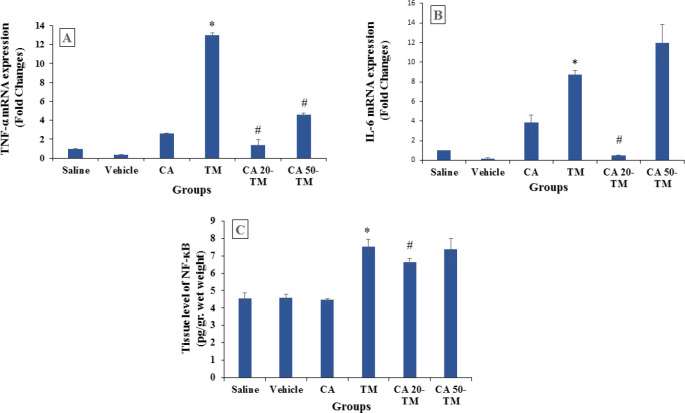
NF-κB level, and *Tnf-α* and *Il-6* gene expression in different experimental groups

The gene expression of NF-κB and two pro-inflammatory cytokines, *Tnf-α* and *Il-6*, in the TM group were markedly increased compared to the Saline group (*P*<0.05) (Figure 4A-C). However, pretreatment with CA lowered the level of inflammatory cytokines compared to the TM group (*P*<0.05). In addition, the treatment with different concentrations of CA has various outcomes in *Tnf-α*, and *Il-6* levels as CA (20 mg/kg) significantly downregulated the expression of these pro-inflammatory cytokines compared to the high concentrations of CA (*P*<0.05).

***CA pretreatment affects the apoptosis in liver tissue***

**Figure 5:**
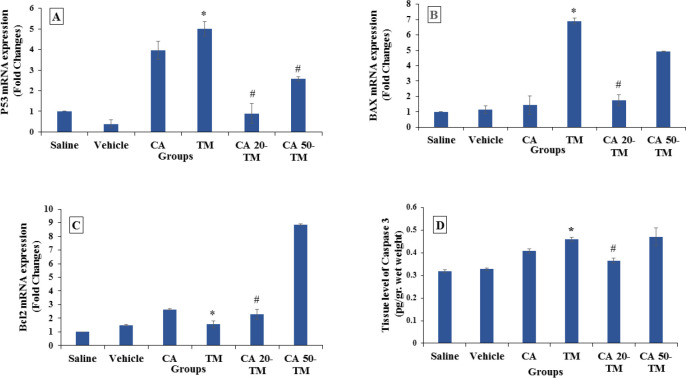
*p53*, *Bax*, and *Bcl2* gene expression and Caspase 3 level in different experimental groups

Real-time RT-PCR results depicted that TM markedly increased hepatocyte mRNA expression of *p53* compared to the Saline group, while this marker was significantly reduced in CA (20 or 50 mg/kg) compared to the TM group (*P*<0.05) (Figure 5A). *Bax* gene expression was also significantly upregulated in the TM-induced mice compared to the saline group (*P*< 0.05), while the CA administration markedly downregulated it compared to the TM group (Figure 5B). 

*Bcl2* gene expression slightly increased in the TM-induced mice compared to the Saline group, and the CA administration markedly raised it compared to the TM group (*P*<0.05) (Figure 5C). Moreover, the expression of apoptotic indexes (*Bax* and *Bcl2*) in the CA 50-TM group increased significantly compared to the Saline group (*P*<0.05). The ratio of *Bax*/*Bcl-2* mRNA expression significantly increased in the TM group in comparison with the Saline group and strikingly decreased in the CA pretreated groups. The ELISA results also showed a marked increase of Caspase 3 level in the TM group compared to the Saline group (*P*<0.05). Nevertheless, CA pretreatment significantly decreased the Caspase 3 level in the CA 20-TM group compared to the TM group (*P*<0.05) (Figure 5D). 

**Table 1:** Primer sequences used in quantitative RT-PCR for C57/BL6 mice model

Gene	primers sequences (Forward (top), Reverse (bottom))
*Grp78*	5′‐*TGTGTGTGAGACCAGAACCG*‐3′ 5′‐*TAGGTGGTCCCCAAGTCGAT*‐3′
*Ire-1*	Forward: 5′‐CACTGCCTGAGACCTTGTTGT‐3′ Reverse 5′‐TTAAAGTCCACTTGATGGAGCC‐3′
*Il-6*	Forward: 5′‐ *TCTGAAGGACTCTGGCTTTG*‐3′ Reverse 5′‐* GATGGATGCTACCAAACTGGA*‐3′
*Tnf-a*	Forward: 5′‐*AGGGTCTGGGCCATAGAACT*‐3′ Reverse 5′‐*CCACCACGCTCTTCTGTCTAC*‐3′
*Perk*	Forward: 5′‐GGCTTGAAAGCAGTTAG‐3′ Reverse 5′‐GGACAGTTGCCTTACAGA‐3′
*p53*	Forward: 5′‐GCCATGGCCAT CTACAAGAA‐3′ Reverse 5′‐CTCGGGTGGCTCATAAGGTA‐3′
*Bax*	Forward: 5’-AGACAGGGGCCTTTTTGCTA-3’ Reverse: 5’-AATTCGCCGGAGACACTCG-3’
*Bcl-2*	Forward: 5’-CTTTGAGTTCGGTGGGGTCA-3’ Reverse: 5’-AGTTCCACAAAGGCATCCCA-3’
*Gapdh*	Forward: 5’-TGGCCTTCCGTGTTCCTAC-3’ Reverse: 5’-GAGTTGCTGTTGAAGTCGCA-3’

GRP78: Glucose regulated protein 78, IRE-1: Inositol-requiring enzyme type 1, IL-6: Interleukin 6 , TNF-α: tumor necrosis factor alpha, PERK: protein endoplasmic reticulum kinase, p53: Tumor protein P53, Bax: Apoptosis regulator, Bcl-2: B-cell lymphoma 2, GAPDH: Glyceraldehyde 3-phosphate dehydrogenase

CA: Chlorogenic acid, TM: Tunicamycin, CA 20-TM: a single dose of 20 mg/kg Chlorogenic acid 60 minutes before Tunicamycin administration, CA 50-TM: a single dose of 50 mg/kg Chlorogenic acid 60 minutes before Tunicamycin administration, GRP78: Glucose regulated protein 78, IRE-1: Inositol-requiring enzyme type 1, PERK: protein endoplasmic reticulum kinase

**Table 2:** Liver enzymes levels in different experimental groups in blood serum samples of C57/BL6 mice model

	**TC (U/L)**	**TG (mg/dL)**	**ALT (U/L)**	**AST(U/L)**	**ALP (U/L)**
	91 ± 4.72	95 ± 2.08	41.33 ± 1.76	147 ± 7.94	9.33 ± 0.88
	87 ± 7.35	147 ± 14.57	37.33 ± 4.37	88 ± 7.93	18.23 ± 2.02
CA	78 ± 0.33	146 ± 5.03	24.66 ± 1.76	124 ± 15.64	16.24 ± 2.33
TM	30 ± 3.05^*^	49.33 ± 9.33^*^	100.6 ± 11.05^*^	366 ± 87.4^*^	35.66 ± 3.48^*^
CA 20-TM	7.33 ± 1.2^#^	46 ± 3.21	40.33 ± 4.63^#^	124 ± 6.43^#^	9.31 ± 0.66^#^
CA 50-TM	6 ± 1.15^#^	29.33 ± 0.88^#^	31 ± 1.52^#^	160 ± 11.26^#^	5 ± 0.57^#^

Mean ± SEM, n = 6, *P<0.05 compared to the Saline Group. #*P*<0.05 compared to the TM group (one-way ANOVA followed by Tukey’s *post hoc* test)

TC: Total cholesterol, TG: Triglyceride, ALT: alanine aminotransfera, AST: aspartate aminotransferase, ALP: alkaline phosphatase, CA: Chlorogenic acid, TM: Tunicamycin, CA 20-TM: a single dose of 20 mg/kg Chlorogenic acid 60 minutes before Tunicamycin administration, CA 50-TM: a single dose of 50 mg/kg Chlorogenic acid 60 minutes before Tunicamycin administration

CA 20-TM: a single dose of 20 mg/kg Chlorogenic acid 60 minutes before Tunicamycin administration, CA 50-TM: a single dose of 50 mg/kg Chlorogenic acid 60 minutes before Tunicamycin administration

Mean ± SEM, n = 6, *P<0.05 compared to saline. #*P*<0.05 compared to TM (Tunicamycin) group (one-way ANOVA followed by Tukey’s *post hoc* test)

CA: Chlorogenic acid, TM: Tunicamycin, CA 20-TM: a single dose of 20 mg/kg Chlorogenic acid 60 minutes before Tunicamycin administration, CA 50-TM: a single dose of 50 mg/kg Chlorogenic acid 60 minutes before Tunicamycin administration, SREBP: sterol regulatory element binding protein, PPAR: peroxisome proliferator-activated receptor, FAS: Fas Cell Surface Death Receptor

Mean ± SEM, n = 6, Pg/gr wet weight, *P<0.001, *P*=0.0, and *P*<0.001 compared to the Saline group. #*P*<0.05 compared to TM (Tunicamycin) group (one-way ANOVA followed by Tukey’s *post hoc* test)

CA: Chlorogenic acid, TM: Tunicamycin, CA 20-TM: a single dose of 20 mg/kg Chlorogenic acid 60 minutes before Tunicamycin administration, CA 50-TM: a single dose of 50 mg/kg Chlorogenic acid 60 minutes before Tunicamycin administration, NF-κB: Nuclear factor kappa-light-chain-enhancer of activated B cells, IL-6: Interleukin 6, TNF-α: tumor necrosis factor alpha

Mean ± SEM, n = 6, Pg/gr wet weight, *P<0.01, *P*= 0.0, and *P*<0.01 compared to the Saline group. #*P*<0.05 compared to TM (Tunicamycin) group (one-way ANOVA followed by Tukey’s *post hoc* test)

CA: Chlorogenic acid, TM: Tunicamycin, CA 20-TM: a single dose of 20 mg/kg Chlorogenic acid 60 minutes before Tunicamycin administration, CA 50-TM: a single dose of 50 mg/kg Chlorogenic acid 60 minutes before Tunicamycin administration

## Discussion

This study showed that CA could reduce liver steatosis and inflammation, plasma ALT and AST, and liver ALP levels. It also decreased gene expression of apoptosis pathways such as *p53*, Caspase 3, *Bax*, and *Bcl-2* in TM-challenged mice. Additionally, CA attenuated pro-inflammatory cytokines, including *Tnf-α* and *Il-6*, and inhibited the nuclear translocation of NF-κB. It also lowered the expression of *Srebp-1c* and *Fas *genes and increased *Ppar-α* gene expression in the TM-induced mice. To the best of our knowledge, this is the first study evaluating the effects of CA on hepatic ER stress-induced steatosis, inflammation, and apoptosis in an animal model.

Recent studies have shown that TM, a common pharmacological ER stressor, can induce ER stress in the hepatocytes and lead to hepatic steatosis (33-35). In this study, we first evaluated the expression of representative ER stress markers. As expected, the mRNA expressions of *Grp78*, *Ire-1*, and *Perk* significantly increased in the TM group. The present study showed that the administration of 20 mg/kg CA provided a marked decrease in the expression of genes related to ER stress. Wang *et al.* (36) have stated that CA decreased *Grp78*, *Perk*, and *Ire-1 *expressions and improved pulmonary fibrosis after bleomycin administration. CA also downregulated ER stress markers in the palmitic acid-induced hepatocytes (37). These reports are consistent with our results, and it seems that CA administration could decrease *Grp78*, *Ire-1*, and *Perk* and thereby ameliorates hepatic ER stress. Surprisingly, in our study, 50 mg CA increased ER stress indexes and upregulated *Grp78*, *Perk*, and *Ire-1* expressions. In this regard, another study showed that intravenous injection of a high dose of CA (49mg/kg/day) increased the number of adherent leukocytes, generation of peroxides in the venular walls, and induced albumin leakage from mesentery venules in the small intestine. Upregulation of inflammatory cytokines and inflammation was also observed in this dose of CA (38). Here, it seems that 50 mg CA increased ER stress and was toxic to liver tissue. 

Our histological results showed severe steatosis (excessive lipid droplet accumulation in hepatocytes), cellular ballooning, and lobular inflammation after thirty hours of TM challenge, based on many previously published (34, 39) and 20 mg/kg CA supplementation prevented TM-induced lipid accumulation, cellular ballooning, lobular inflammation. However, administration of a high dose of CA (50 mg/kg) cannot improve liver steatosis and inflammation in ER-stress-induced mice. The histological results were compatible with those obtained from the expression of genes related to ER stress and confirmed the positive effect of CA in preventing ER stress-induced liver injuries. 

In accordance with our histopathological investigation, Shi *et al.* suggested that CA could alleviate the cadmium-exposed chicken livers. They showed that the liver histopathology and ultrastructure of hepatocytes were improved after the poisoned chickens were treated with α-lipoic or CA (40). 

It is notable that the hepatic function and histology were significantly improved after the suppression of oxidative stress by CA treatment in ischemia/reperfusion injury in rat liver, as designated by hepatic structure improvement (41). 

This study showed that TM administration induced TG accumulation in the hepatocytes and led to steatosis by upregulating the *Srebp-1c* and *Fas* gene expression and downregulating the *Ppar* gene expression in the mice. However, CA alleviates steatosis in liver tissue by downregulating the expression of *Srebp-1c* and *Fas* genes and upregulating the *Ppar* gene expression in TM-induced ER stress. In accordance with our results, Li and his colleagues showed that in bovine hepatocytes, SREBP-1C overexpression could induce TG accumulation by increasing lipid synthesis and decreasing lipid oxidation. Moreover, SREBP-1c overexpression upregulated the expression of other genes involved in TG synthesis, including FAS (54). 

The anti-obesity effect of *Nostoc commune* ethanol extract could downregulate the mRNA expression of adipogenesis, including *PPAR-γ* and *SREBP-1c* and lipid lysis-related genes in epididymal adipose tissue (55). It seems that CA administration could alleviate liver steatosis through *Srebp-1c* and *Fas* downregulation, and Ppar-a upregulation. It seems that CA administration through stress attenuation downregulated *Srebp-1c*, and following that, the Fas enzyme also upregulated *Ppar-α*, and afterward, liver steatosis was alleviated.

In this study, NF-κB protein levels, together with TNF-α and IL-6, increased in mice with ER stress. Previous studies have shown that NF-κB protein levels and the expression of inflammatory genes were upregulated in ER stress model (42, 43). Aslan *et al.* (35) established that serum levels of ALT, AST, and ALP markedly rose in TM-induced ER stress in male rats. Compatible with previous reports, 20 mg/kg CA effectively declined NF-κB levels and IL-6 and TNF-α expression and attenuated TM-induced hepatic inflammation.

In several documents, it has been shown that some concentrations of CA can attenuate inflammation (28, 44) while some other concentrations can provide stimulatory effects on proinflammatory cytokines such as interleukins and TNF-α (41, 45). Our results showed that 50 mg/kg CA increased NF-ĸB, *Il-6*, and *Tnf-a*. Anqi *et al.* (46) have reported that 40 mg/kg CA increased IkB-α and induced apoptosis in breast cancer tumors. Herein, it seems that a high dose of CA could not decrease inflammatory markers due to the inability of ER stress attenuation.

It has been reported that ER stress induces apoptosis in many diseases (47, 48). In this study, CA decreased gene expression of tumor suppressor *p53*, Caspase 3, and pro-apoptotic *Bax* and increased anti-apoptotic *Bcl-2* in the liver tissue of TM-challenged mice. A study demonstrated that CA decreased caspase 3, 9, and 12 in RLE-6TN cells and pulmonary tissue of mice with ER stress (49). Another study proved that CA induced cell apoptosis in ER stress provoked by palmitic acid (37). Moreover, the administration of CA had anti-apoptotic and antifibrogenic effects showing that it can be used as a beneficial treatment for various liver diseases (50). Although the present study demonstrated that the use of 20 mg/kg CA inhibits apoptosis, a higher concentration of CA has a contrary effect and induces the expression of apoptosis-related genes; So, the effect of CA on the expression of genes involved in the apoptosis mechanism is probably dose-dependent (50). In our previous study, a high concentration of CA upregulated the expression of apoptotic genes such as *p53*, *Bax*, and *caspase‐3* in mice with breast cancer tumors (51). Therefore, CA in low concentrations possibly shows a protective effect against TM-induced ER stress in the liver tissue, and in high concentrations, it could be useful for cancer treatment by induction of apoptosis. In recent years, several studies have revealed that CA plays an important role in tumor prevention. CA can decrease the proliferation of A549 human lung cancer cells (52) and suppress glioma growth by repolarizing the phenotype of macrophages (53). It also induces apoptosis in MCF-7 and MDA-MB-231 breast cancer cell lines in a dose-dependent manner and disrupts the cell cycle (54).

## Conclusion

Our study demonstrated that TM treatment resulted in liver ER stress and low concentration of CA can improve steatosis, and hepatic inflammation and plays an important role in inhibiting ER stress symptoms. Also, 20 mg/kg of CA decreased lipid metabolism-related transcriptional factors, enzyme expression, and apoptosis agents in ER stress-induced mice. However, high concentrations of CA had negative effects on steatosis, inflammation, and apoptosis. Therefore, choosing a suitable concentration of CA is critical for the treatment of liver injuries. 

## Authors’ Contributions

AM designed the experiments; TKM performed experiments and collected data; MA discussed the results and strategy; FH Supervised, directed, and managed the study; AM, TKM, MA, and MG approved the final version to be published.

## Conflicts of Interest

The authors declare that they have no known competing financial interests or personal relationships that could have appeared to influence the work reported in this paper.
